# A Path Analysis Evaluating the Impacts of Childhood and Adult Trauma on Mental Health Outcomes at Two Psychiatric Hospitals in Johannesburg, South Africa

**DOI:** 10.1192/j.eurpsy.2023.1149

**Published:** 2023-07-19

**Authors:** M. Galvin, A. Scheunemann, A. Kim, A. Moolla

**Affiliations:** University of the Witwatersrand, Johannesburg, South Africa

## Abstract

**Introduction:**

Like many under-resourced settings, there is a large gap between burden of mental illness and availability of services in South Africa. Because South Africa also bears a high burden of adverse childhood events (ACEs) and adult trauma, mental illness is often preceded in individuals by either or both. While studies within South Africa have examined the association between ACEs and distress in adulthood and adult trauma and adult mental distress, there is less knowledge of how these preceding factors interact to affect mental distress together, particularly in clinical populations.

**Objectives:**

Using path analysis, this study seeks to ascertain the impact that ACEs and adult trauma have on mental illness in urban South Africa. Understanding the perceptions and experiences of people living with mental illness is key not only to expanding biomedical services and ensuring appropriate and effective mental health treatment, but can also help identify ways to prevent mental illness in the future.

**Methods:**

This study uses data collected from 309 psychiatric out-patients at two public psychiatric hospitals in Johannesburg. Ethics approval was received and data were collected in-person between January and June of 2022. Patients 18 years and above, of African descent, and willing to provide informed consent were invited to participate. The survey included questions about demographics COVID-19, adverse childhood events, adult traumatic events, depression, anxiety, and stress. Participants were also invited to take part in a brief, semi-structured interview. Data were analyzed via path analysis, using the lavaan package in R, version 4.1.1.

**Results:**

Incidences of both ACEs and adult trauma were significantly associated with three mental illness outcomes – depression, anxiety, and stress. An aggregated adult trauma score was found to partially mediate the association between total ACEs and depression, anxiety, and stress. When analyzed separately, total adult trauma partially mediated the association between ACEs including childhood verbal abuse, sexual abuse, emotional neglect, and mental illness in the household and depression, anxiety, and stress. Total adult trauma also partially mediated the association between childhood physical abuse and depression and anxiety, but not stress.

**Conclusions:**

This study highlights the importance of dis-aggregating adverse childhood events when exploring their effects, while also reinforcing previous findings that ACEs increase the likelihood of experiencing adult trauma and mental illness. Future studies should attempt to pinpoint which ACEs are most impactful, and target those in particular for prevention in childhood and intervention in adulthood, to mitigate their deleterious impacts.

**Disclosure of Interest:**

None Declared

